# Rose Pearl

**Published:** 1868-11

**Authors:** C. M. Wright


### ROSE PEARL.

Considerable quiet interest has been felt, and many inquiries made by numbers of Western Dentists about the new base —the new material idea—introduced to the profession by Dr. McClelland, of Louisville, under the name of Rose Pearl. At two or three Dental societies Rose Pearl has made its modest bow, and eastern men have made further acquaintance with it, and are rapidly becoming its firm and fast friends. Western Dentists are, at present, almost strangers, and consequently ignorant of its many good and agreeable qualities when introduced into practice, and into the mouths of patients. As I have recently had opportunities for cultivating an acquaintance, with and I may say a strong friendship for Rose Pearl, and as many of my friends in the Dental profession, who are, of course, readers of the REGISTER, have requested my opinion of the new base, perhaps I may be permitted to occupy a very little space in these columns, in behalf of this really valuable base for artificial teeth. Many have seen some of the earlier specimens of the work, and know that plain teeth are used, and that the base and gum are one continuous piece. This, of course, is an advantage that every operator acknowledges, for he now has room for the display of his abilities as an artist, in imitating nature, by causing slight irregularities, and by arranging the teeth to harmonize with the contour of the face. He is not bound by sections or by single gums, and his operations for restoring the natural teeth are not so confined, and good taste need not be violated as it so frequently has been, and must be in full dentures on gold, rubber and other similar plates. Different shades of gum color can be selected, and the gum carved to suit the necessity of a case for expression, and to please the operator, and in prominent natural gums, where but very little material can be used on account of the full appearance given to the lips this material is particularly well adapted because of its strength.

I have recently had a case where the prominence of the superior gum and the difficulty of obtaining a correct occlusion of the anterior teeth prevented the employment of gum teeth, mounted on other plates, and where the Rose Pearl was used for plate and gum. The result was particularly pleasing.

The material itself is fine grained semi-translucent, elastic and inoderous, and can be successfully manipulated by any Dentist who can make good gold work.

There are some, no doubt, who call themselves Dentists, and who are now professing to practice this specialty, having had fully six weeks instruction in working rubber, who will not be able to succeed in producing good or practical pieces with this material. It is not a work that will please mechanical bunglers, nor indeed any but those in the profession who have taste and ability, and who are really first-class Dentists. To such the new base will be, and in the East is being received and cultivated with a degree of enthusiasm that is quite refreshing, and is proving contagious.

I have been rather conservative in practice—have hesitated before taking up new things—fearing constitutionally to be humbugged, and even after working Rose Pearl in the laboratory and becoming somewhat fascinated with the method of construction, would not cordially endorse it for practicability till compelled by the perfect success, the beautiful results in many practical sets of artificial teeth, and the expressions of satisfaction and pleasure that patients have given in regard to it. For full sets of teeth there is nothing equal to Rose Pearl in a majority of cases. While it has the advantages of continuous gum and porcelain work, it is lighter in weight, and must be preferable to the patient, and it can be repaired and changed to suit the shrinkage of the alveolar ridges by absorption, with facility. It is perfectly cleanly and tasteless, as a suspicious gentleman of this city can aver, for before permitting a set to be made for him he carried a small piece of the material in his mouth for a week to "see whether it tasted." "He was also satisfied that it would not soften down." The fluids of the mouth do not act upon it at all.

I will not intrude on valuable space further than to add that with Rose Pearl a perfect *fit* can be obtained, as it is capable of close adaptation to the mucus membrane, and sharp and distinct impressions of the rugæ are obtained.

I have not written this to persuade any one to try this base, nor am I interested in its acceptance by the profession, or my friends, but I have only desired to answer inquiries, and expressed my opinion, based on experience with and judgment of it—to add my testimony in its favor for the benefit of those who are inquiring into its merits.

C. M. Wright.

